# A Review of Digital Health Strategies in 10 Countries With Young Populations: Do They Serve the Health and Wellbeing of Children and Youth in a Digital Age?

**DOI:** 10.3389/fdgth.2022.817810

**Published:** 2022-03-17

**Authors:** Louise Holly, Robert Dean Smith, Njide Ndili, Christian Franz, Enow Awah Georges Stevens

**Affiliations:** ^1^The Lancet and Financial Times Commission on Governing Health Futures 2030 Secretariat, Global Health Centre, Graduate Institute, Geneva, Switzerland; ^2^PharmAccess Foundation, Lagos, Nigeria; ^3^CPC Analytics, Berlin, Germany; ^4^Hadassah Medical Centre, Yaounde, Cameroon

**Keywords:** digital health, digital health strategies, digital health governance, children, youth, Africa

## Abstract

Children and youth merit special attention from digital health policymakers and practitioners because of the great potential for digital transformations to both enhance and undermine their health and wellbeing. However, an analysis of digital health strategies from 10 African countries with young populations suggest that national approaches to digital health are overlooking young people's specific health needs and unique risks in relation to digital technologies and data. To better serve the needs of children and youth in a digital age, future digital health strategies—and the global guidance that many strategies are based upon—should consider the ways in which digital transformations can positively or negatively impact the health and wellbeing of different populations, and the forms of cross-sectoral and multi-stakeholder collaboration required to amplify or mitigate them. Future strategies should be developed through inclusive processes that support young people's right to participate in decision-making that affects their lives.

## Introduction

In October 2021, a *Lancet* and *Financial Times* Commission titled Governing health futures 2030: Growing up in a digital world^1^ published a report with recommendations for a value-based approach to governance of digital transformations that maximizes health and wellbeing, particularly for current and future generations of children and youth (1). Children and youth are a group that merits attention from digital health policymakers and practitioners because of the potential that digital transformations offer for countries to improve newborn, child, adolescent, and youth health. Furthermore, in many countries, young people are the most digitally connected and literate group and as such have greatest exposure to the potential opportunities and risks in relation to the use of digital technologies. Considering children and youth in the governance of digital health—which includes not only the application of digital technologies for health but the broader ways in which digital transformations can affect health—is therefore essential to optimize the health benefits of digital transformations for current and future generations.

In this review, we analyze national digital health strategies from 10 African countries with large and growing populations of children and youth. National digital health strategies are seen as essential tools for governments to set out their priorities and align governance aims for digital transformations in health (2). We begin by analyzing to what extent children and youth are represented in the strategies and then analyze the inclusion of universal health coverage (UHC) and core principles that we argue should be prioritized within digital health governance in order for young people to flourish in a digital age.

To understand barriers to implementation of these digital health strategies we conducted interviews with digital health experts from the 10 countries. Together, our analysis and interview findings suggest that digital health strategies do not sufficiently serve the needs of children and youth in a digital age. We propose ways for policymakers to strengthen the content and implementation of national digital health strategies so that they can better serve young people's health and wellbeing. Specifically, our recommendations focus on including children and youth in the development of national digital health strategies, and for strategies to consider the wider effects of digital transformations on health more thoroughly.

## Background: National Digital Health Strategies, Children and Youth, and Governance

“Digital health” is an umbrella topic as exemplified by the WHO's *Global Strategy on Digital Health*: “Digital health expands the concept of eHealth to include digital consumers, with a wider range of smart and connected devices. It also encompasses other uses of digital technologies for health such as the Internet of Things, advanced computing, big data analytics, artificial intelligence including machine learning, and robotics” (2). Therefore, the governance of digital health includes both the governance of digital technologies for health as well as the governance of the ways in which the broader digital transformations influence health outcomes.

While digital health appears promising for accelerating progress toward UHC and other health goals, it comes with both opportunities and challenges. Opportunities include increased access to health care, better health outcomes, and increased efficiency of health systems. Risks include further widening health inequities due to the digital divide and biases within AI-powered health solutions, the proliferation of unregulated digital health tools, increased exposure to health misinformation, and threats to health information privacy and security (3).

### Children and Youth Online

Children and youth are amongst the groups most affected by digital transformations. First, global estimates of internet users by age groups across geographies show that young people use the internet more than the overall population: While nearly 70% of people aged 15–24 are online, the average in the total population is just 48% (4). Although connected, children and youth often lack digital literacy and skills to protect themselves from online harms, predatory marketing and data extraction practices, infringements on consent, and other rights violations (5, 6). Data on young people's disproportionately high use of digital technologies often hides the fact that many children's and youth's online access is irregular and of poor quality, and many still remain totally unconnected. According to UNICEF and ITU, two out of three children globally have no broadband internet connection at home (7). Children and youth that are digitally disconnected are at increased risk from material and social deprivations resulting from reduced access to education, job opportunities, healthcare, and social environments for mental wellbeing.

### Challenges to Implementing Digital Health Governance for Children and Youth

A focus on children and youth within digital health governance has begun to emerge in other recent initiatives. For example, the WHO recently published guidelines on *Youth-Centered Digital Health Interventions* (8). As such, attention has increased on the way that digital transformations influence young people's health and wellbeing, and the need for governance structures that aim to enhance children's and youth' health and rights (9).

However, digital health governance poses novel challenges because of the ways that digital transformations intersect across traditional domains of governance. For example, digital platforms and their content may be supportive or damaging to health, but their governance does not fall within the remit of health ministries. While a social determinant of health such as education may be addressed within a Ministry of Education, digital determinants of health such as connectivity and access to quality online health information often requires inter-ministerial coordination.

Further complicating digital health governance is the need for governments to establish meaningful and ongoing child and youth engagement to understand the perspectives of young people. While some government departments may be able to report particular examples of youth engagement on digital health, continuing engagement of children and youth in the governance of digital transformations and health is rare or even non-existent (8, 10). Because of the novel and evolving nature of digital transformations, governments are often underprepared to govern, resulting in judicially Reactive governance in cases of human rights and child rights violations vs. legislatively proactive governance (11).

To establish more proactive governance that maximizes the opportunities of (digital) health, the WHO and ITU have encouraged all countries to develop national digital health strategies and have provided a toolkit to assist countries in constructing these strategies (12). WHO encourages countries to actively use digital health strategies to accelerate progress toward UHC and other health-related targets of the Sustainable Development Goals, and to develop these strategies through an inclusive multi-stakeholder approach (2). Existing global guidance does not specifically recommend that countries design and implement national digital health strategies that fully reflect the needs and voices of children and youth. A review of national digital health strategies was therefore undertaken to understand the extent to which these strategies reflect young people's specific health needs and support their right to participate in decision-making that affects their lives.

## A Review of National Digital Health Strategies in Ten African Countries With Large Youth Populations

We collated and reviewed national digital health strategies from around the world in order to better understand countries' priorities and activities for strengthening digitally-enabled health systems, barriers to implementation, and the extent to which the needs and views of young people—who stand to inherit the health systems that are being digitally-transformed today—have factored in these efforts.

This article focuses on a deeper analysis of digital health strategies from African countries where young people under 25 make up a substantial proportion of the population. There were three reasons to focus on African digital health strategies: Firstly, Africa is home to the countries with the highest proportions of young people, and it is estimated that by 2030 almost one-third of the world's children will live in Africa (13). Secondly, both disease burden and levels of mortality are high among children and youth in Africa (14). Finally, whilst the percentage of young people under 25 with internet access at home is currently low-−5% in West and Central Africa; 13% in East and Southern Africa—the number of African children and youth coming online will increase rapidly over the coming decade (7).

### Search Strategy and Selection Criteria

**Figure 1:**
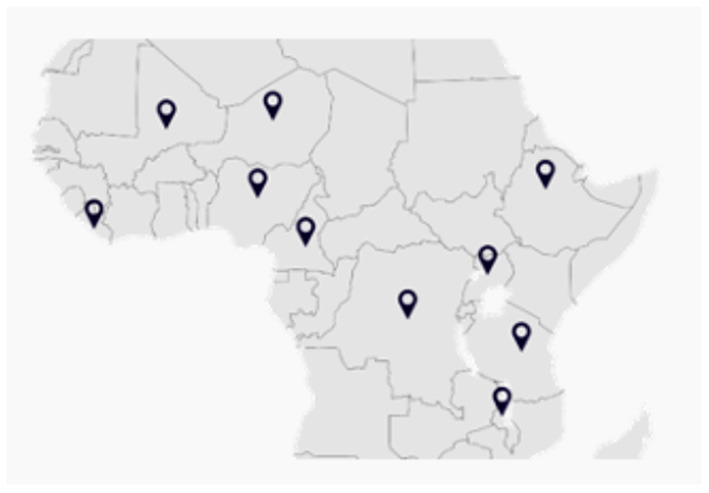
Selected countries for the study.

Ten countries with large child and youth populations were selected for the purposes of the study. This included: Cameroon, Democratic Republic of the Congo (DRC), Ethiopia, Liberia, Malawi, Mali, Niger, Nigeria, Tanzania, and Uganda (see Figure 1; Table 1). These countries were also found to be of interest because they have significantly higher uptake of digital technologies among young people; for example, 39.6% of 15–24-year-olds were using the internet vs. 28.6% in the overall population of Africa (15).

**Table 1:** Population under 25 years by country.

	**As % of total**	**In millions**
Niger	69.2	16.7
Uganda	67.0	30.6
Mali	66.9	13.5
DRC	65.0	58.3
Malawi	64.0	12.2
Tanzania	63.1	37.7
Nigeria	62.9	129.6
Cameroon	61.9	16.4
Ethiopia	61.4	70.6
Liberia	60.4	3.1

*Source: (13). World Population Prospects*.

The WHO's *Directory of eHealth Policies*^2^ was our starting point for searching national digital health strategies for the 10 focus countries of this review. We then used internet searches to identify more current strategies. The WHO Regional Office for Africa and key informants interviewed for this study also provided copies of digital health strategies that were not available online.

### Overview of National Digital Health Strategies

To analyze the strengths and limitations of national digital health strategies and their implications for children and youth, we collected information on six domains. This included (i) the period of validity of the strategy and which government ministry it was created by; (ii) the focus and goals defined within the strategy; (iii) how children and youth are considered within the strategy, if at all; (iv) the alignment of strategies to UHC goals; and (v) other core values or principles represented in the strategies. The former three domains were meant to provide descriptive information for the purposes of this study, while the latter two domains aimed to understand the normative basis for the strategies.

#### The Current Status of National Digital Health Strategies

All 10 countries have published a strategy to guide the digital transformation of their health system as shown in Table 2. In most cases, these strategies were developed to support the implementation of a national health strategy and as part of a broader digital transformation agenda. Nine out of the ten countries had current strategies at the time the research was conducted in the third quarter of 2020. During the research period, consultations were underway in Malawi and Mali to develop a new digital health strategy, but drafts were not available to review.

**Table 2:** Overview of digital health strategies.

	**Strategy**	**Period of validity**	**Lead ministry/agency**
Cameroon	National Digital Health Strategic Plan	2020–2024	Ministry of Public Health
DRC	Plan National de Développement de l'Informatique de Santé	2020–2024	L'Agence Nationale de l'Ingénierie Clinique, de l'Information et de l'Informatique Sanitaire (ANICiiS), Ministère de la Santé Publique
Ethiopia	Information Revolution Roadmap II	2020–2029	Ministry of Health
Liberia	Health Information System and ICT Strategic Plan	2016–2021	Ministry of Health
Malawi	Monitoring, Evaluation and Health Information Systems Strategy	2017–2021	Ministry of Health and Population
Mali	Politique National Cybersanté	2013	Agence Nationale de Télésanté et d'Informatique Médicale (ANTIM)
Niger	Stratégie Nationale E-Santé	2019–2023	Ministère de la Santé Publique
Nigeria	National Health ICT Strategic Framework	2015–2020	Federal Ministry of Health
Tanzania	National Digital Health Strategy	2019–2024	Ministry of Health, Community Development, Gender, Elderly and Children
Uganda	National eHealth Strategy	2017–2021	Ministry of Health

The titles of the 10 strategies reflect changing terminology. More recently published strategies, such as ones from Cameroon and Tanzania, are called “digital health” strategies. Whereas, Mali, Niger and Uganda have “eHealth” strategies and the remaining are health “information/informatics” or “ICT” strategies.^3^

#### The Focus and Goals of Digital Health Strategies

As seen in Table 3, all 10 strategies reflect their respective government's aspiration to use digital technologies and data to improve the performance of health systems and achieve better health outcomes for the population. The situation analysis within each strategy describes both significant health challenges and relatively low levels of digital maturity. Therefore, all strategies place strong emphasis on building the necessary foundations for digital transformations in health. With the exception of Mali, all strategies draw heavily on the WHO-ITU's *National eHealth Strategy Toolkit* and many strategies are structured according to the Toolkit's seven building blocks.

**Table 3:** Strategy scope and aspirations.

	**Strategy focus**	**Overarching goal and objectives**
Cameroon	A national framework for the development of digital health services over 5 years to improve health promotion, disease prevention, case management, health system strengthening and governance, and strategic management of the health system. The strategy seeks to build digital maturity across all the eHealth building blocks.	Vision: By 2024, digital health will effectively contribute to UHC through informed decision-making at all levels of the health pyramid, and through reliable, robust, secure, and interoperable systems. General objective: By 2024, improve the performance of the health system through optimal use of effective digital technologies at all levels of the health pyramid.
DRC	The strategy is narrower in scope than previous versions and is limited to a selection of essential applications that can contribute substantially to the achievement of the national objectives of UHC. These are: standardization and interoperability of IT solutions deployed in the health sector; strengthening health workforce capacities through remote learning and decision-support tools; building ICT infrastructure of health facilities; implementation of a national HIS; and improved governance of eHealth.	Vision: To create an integrated HIS, informed by first-line digital tools focused on the patient and health professionals, offering a complete source of information in a reliable, accessible and timely manner for steering health policy toward UHC. Goal: Implementation of the strategy contributes to the DRC's health and development objectives.
Ethiopia	As part of a broader “Information Revolution” agenda, the focus of this roadmap is to guide a radical shift from traditional methods of data utilization within the health sector to a systematic information management approach powered by a corresponding level of technology. The main strategies identified to achieve this are: enhance informed decision-making; improve data quality; enhance digital health information technology; improve HIS governance and leadership.	Vision: A strong HIS that produces high quality data and being the credible source of the health information. Mission: To advance and transform in data generation, analysis, synthesis and sharing quality data through nurturing digital information technology to promote data demand and informed decision-making at all levels of the health system.
Liberia	The strategic plan focuses on strengthening HIS to create a comprehensive and interoperable health information system that leads to improved health outcomes. Improving internet coverage, strengthening ICT infrastructure and increasing skilled staff are identified as necessary steps for delivering on the actions in the plan.	Vision: The Health System of Liberia is supported by a comprehensive and interoperable HIS, leading to improved health outcomes for individuals and communities in Liberia. Goal: By 2021, the National HIS of Liberia will produce quality data and information used in support of the health system functions at all levels, with a solid governance and management structure, using appropriate information and communication technology, including data confidentiality and security, at an affordable cost.
Malawi	The strategy's aim is to strengthen HIS and fully transition from paper-based to electronic systems at the point of care to leverage the power of ICT in the generation of real-time decision support data. An interoperability layer will be developed to allow data to be linked and shared across all parts of the health information management system.	Vision: A sustainable, integrated national HIS capable of generating and managing quality health information for supporting evidence-based decision making by all stakeholders at all levels of the health system. Objectives: To strengthen the health sector's capacity to use data for decision making; and to ensure that HSSP II is adequately monitored with high-quality data that are routinely reported, analyzed, and disseminated.
Mali	The strategy outlines how eHealth can support the objectives of the country's 10-year Health and Social Development Plan (PDDSS). The creation of a national digital health network is a high priority to allow the interconnection of health facilities and facilitate the exchange of secure health data. Increasing connectivity of health facilities; modernizing the HIS; strengthening human resources for health and ICT specialists; and developing guidelines on standards and interoperability are all planned activities to support the effective development and provision of eHealth services.	Vision: By 2030, ICT will be used at all levels of the health system to make reliable, secure and up-to-date health and medical information available, improve the quality of care and its accessibility, and make the management of health structures efficient. Overall objective: Contribute to the improvement of the health system through the inclusive use of information and communication technologies.
Niger	The strategy is structured around two pillars. Firstly, building an environment conducive to the development and use of eHealth services by increasing the connectivity of health structures; increasing the availability of skilled human resources; standardization and interoperability; and strengthening eHealth governance. The second pillar is focused on expanding the provision of eHealth and telehealth services.	Vision: By 2030, ICTs will be used effectively in rural areas and particularly in remote and landlocked areas in order to improve the health of Nigerien populations. Mission: Use ICTs at all levels of the health system to make reliable health and medical information available, improve the quality of care and make efficient management of the resources of the country's health structures.
Nigeria	The strategy provides a vision and guide for the strategic application of ICT and alignment of current investments in technology within the health system toward a digitized health system that will help Nigeria achieve UHC. The strategy outlines steps to build maturity across all the ITU-WHO building blocks and has a strong emphasis on increasing financial coverage for healthcare through use of ICT for health insurance and other health-related financial transactions.	Vision: By 2020, health ICT will help enable and deliver universal health coverage in Nigeria. Intended outcomes of the strategy include improved access, coverage and quality of health services through effective use of ICTs, telemedicine, and HIS.
Tanzania	The strategy builds on earlier versions with a shift in focus from collecting and reporting from aggregate data to client-level data, as well as data use at all levels of the health system. This strategy looks at the application of different technologies for disease prevention and to promote healthy behaviors. It also considers the need to explore innovative approaches and emerging technologies and their potential to support UHC.	Vision: Better health outcomes through a digitally enabled health system. Mission: To accelerate the transformation of the Tanzanian health care system through innovative, data-driven, client-centric, efficient, effective, and integrated digital health solutions.
Uganda	The strategy aims to standardize ICT for health infrastructure and services to ensure that they are aligned to health service requirements, are interoperable, and enable more efficient use of healthcare resources. The strategy outlines an incremental approach from paper intensive processes to the development of electronic health records that will enable the flow of quality and relevant health information and decision-making across the healthcare network. Other pillars of the strategy seek to establish telehealth and mHealth services to deliver healthcare and empower communities; and increase awareness of eHealth through mass campaigns.	Vision: Effective use of ICT for better health outcomes of the Ugandan population. Mission: To transform the health of the people of Uganda by promoting effective utilization of ICT. Goal: To harness and create an enabling environment for the development and utilization of sustainable, ethically sound and harmonized ICT at all levels to promote health and improve health services delivery in Uganda.

Each strategy has a strong emphasis on strengthening integrated health information systems (HIS) to improve data collection and use for decision-making. In the cases of Ethiopia, Liberia and Malawi, HIS is the primary focus of the strategy. In all countries, increasing the availability of high-quality data, and the capacity of the health workforce to use that data, are recognized as essential for optimizing the efficiency and effectiveness of health services.

In addition to strengthening HIS, all strategies outline plans to use telemedicine, mHealth and/or eHealth tools to improve quality and increase service coverage, especially for underserved populations. Tanzania is unique in having a strategy that includes a reference and commitment to explore and research emerging technologies such as artificial intelligence.

#### How Children and Youth Are Considered in Digital Health Strategies

While the national health strategies of all 10 countries prioritize newborn, child, and adolescent health, none of the digital health strategies reviewed included any specific consideration of children and youth in the development and application of digital technologies, the management of health data, or the development or monitoring of the strategy. The context sections of several strategies did reinforce that improved child and adolescent health are intended outcomes of digital health (see Table 4). Ethiopia's strategy identified “youth-focused” as a guiding principle for the health sector but did not elaborate on how this principle would be applied in relation to digital health. Several strategies noted their country's young population and opportunities presented by large numbers of young people entering the workforce to support the digital transformation agenda. However, none of the documents reviewed indicated that children and youth had been involved in the development of the strategy, and none referred to the potential health benefits or concerns arising from broader digital transformations that may impact children and youth.

**Table 4:** Focus on children and youth within digital health strategies.

	**High youth population noted in landscape analysis**	**Presence of young workforce/innovators identified as an opportunity**	**Use of digital health/data to improve child and adolescent health is a priority**	**Children and/or youth as a guiding principle for the strategy**	**Children and youth involved in development of the strategy**
Cameroon	X	X			
DRC		X	X		
Ethiopia				X	
Liberia			X		
Malawi			X		
Mali	X				
Niger	X		X		
Nigeria					
Tanzania			X		
Uganda	X	X	X		

#### Alignment of National Digital Health Strategies to UHC and Its Foundational Values

Improving the health of children, youth, and other left behind communities as part of a primary healthcare approach is recognized to be the cornerstone of UHC (16). Our analysis therefore included an assessment of the extent to which digital strategies look to support the achievement of UHC as represented in Table 5.

**Table 5:** Strategy alignment to UHC.

	**Alignment to UHC**
Cameroon	Strategy's vision is that digital health will contribute to UHC.
DRC	Objective of the strategy is aligned to UHC. Recognizes that the realization of UHC will require eHealth.
Ethiopia	UHC is a guiding principle for the strategy. An objective of stronger HIS is to monitor progress toward UHC.
Liberia	Universal coverage is one of the national values guiding the strategic plan.
Malawi	Strategy supports monitoring of UHC and compliments broader national strategies to achieve UHC.
Mali	Predates global commitments to UHC but is in keeping with national health policy to improve health coverage.
Niger	Strategy reinforces Niger's commitment to UHC.
Nigeria	Strategy seeks to align health ICT with the achievement of UHC.
Tanzania	Strategy proposes that digital health will fast-track the achievement of UHC.
Uganda	Strategy supports the Health Sector Development Plan which has UHC as its goal.

Nine out of 10 strategies are explicitly aligned to the Sustainable Development Goals (SDGs) and the realization of UHC. The exception is Mali's strategy which predates the adoption of the SDGs and high-level political commitments to UHC.

The UHC and SDG agendas are built upon a number of core values including equity, ethics, and human rights (17). Table 6 provides an overview of whether these values are explicitly referenced in a country's digital health strategy, if there are elements of the strategy that support a value or principle and thus indirectly referencing, or if there is no reference to the value.

**Table 6:** References to equity, ethics, and human rights within digital health strategies.

	**Equity**	**Ethics**	**Human rights**
Cameroon	Indirect reference	Explicit reference	No reference
DRC	Indirect reference	No reference	No reference
Ethiopia	Explicit reference	Explicit reference	Indirect reference
Liberia	Explicit reference	Reference	Indirect reference
Malawi	Explicit reference	No reference	Explicit reference
Mali	Explicit reference	No reference	Indirect reference
Niger	Explicit reference	Explicit reference	Indirect reference
Nigeria	Explicit reference	Explicit reference	Indirect reference
Tanzania	Explicit reference	No reference	Indirect reference
Uganda	Explicit reference	Explicit reference	Explicit reference

Eight out of 10 strategies explicitly reference equity as a core principle and the remaining two (Cameroon and DRC) indirectly reference equity by stating that digital health will be used to reduce health disparities through its alignment with an equity-focused national health strategy.

Five strategies explicitly talk about the need for an ethical approach to digital health; one references the need for users of HIS to be trained in ethics (Liberia); and the remaining four do not mention ethics. Only two strategies (Malawi and Uganda) outline a human-rights based approach. Two strategies (Mali and Niger) note that the right to health is enshrined in the country's constitution and one (Nigeria) references the right to privacy. Three strategies (Ethiopia, Liberia and Tanzania) do not use rights-based language but indirectly talk about the need to protect individual privacy and confidentiality. None of the strategies make any specific reference to children's rights.

#### Inclusive Processes to Strategy Development

The inclusion and the enfranchisement of affected communities are widely recognized to be effective strategies for addressing global health challenges and strengthening health governance. In line with the Principles for Digital Development, approaches to digital health should be designed with the user^4^. This means that intended users or beneficiaries should be partners in the design and application of digital health solutions, and as such the principle of inclusion should be promoted within digital health strategies. Further, the Principles indicate that communities—including children and youth—should be enfranchised to contribute to digital health policy and to use digital health tools to support their own health.

As summarized in Table 7, six strategies make no reference to the inclusion of communities. Two (Ethiopia and Uganda) are explicit about the importance of involving communities in planning, implementation, and monitoring. Niger commits to civil society involvement in creating a legal framework. Tanzania refers to the Principles for Digital Development but doesn't reference activities to include communities.

**Table 7:** References to inclusion and enfranchised communities within digital health strategies.

	**Inclusion**	**Enfranchised communities**
Cameroon	No reference	No reference
DRC	No reference	No reference
Ethiopia	Explicit reference	Explicit reference
Liberia	No reference	No reference
Malawi	No reference	No reference
Mali	No reference	Explicit reference
Niger	Indirect reference	Explicit reference
Nigeria	No reference	Empowered health workers
Tanzania	Indirect reference	Empowered health workers
Uganda	Explicit reference	Explicit reference

Four strategies (Ethiopia, Mali, Niger and Uganda) explicitly plan to use digital tools to enfranchise and empower communities. Two strategies (Nigeria and Tanzania) plan to use digital tools to empower health workers and the remaining four strategies make no reference to enfranchisement.

## Discussion: Is an Insufficient Focus on Children And Youth in Digital Health Strategies a Barrier to Their Implementation?

All 10 countries in this study have a digital health strategy but the realization of these strategies is not progressing at the anticipated pace. To better understand the reasons for this, we conducted interviews with digital health experts from the study countries. Through these interviews, we were also able to consult experts on the advantages and disadvantages of focusing on children and youth in national digital health strategies.

### Interview Methodology

Between August and October 2020, we contacted more than 50 digital health experts working in 1 or more of the 10 focus countries *via* email or social media and invited them to participate in an interview. To identify potential interviewees from different sectors—including government; academia; civil society; international organizations; private sector; and donor agencies)—we drew upon known experts in digital health and snowball sampled through these contacts.

Authors' host institutions do not have an ethical approval committee or institutional review board. An interview protocol was developed and shared with participants in advance of their participation in the study. This protocol outlined the purpose of the study and how participant data would be used. The protocol confirmed that interview data would be anonymized and participant privacy would be protected. Oral consent was sought for a second time before recording interviews.

A semi-structured, open-ended questionnaire was shared with respondents in advance and used as an interview guide. Questions focused on implementation progress and bottlenecks in four areas: digital infrastructure; digital health; growing up in a digital world; and governance of data in health (see Box 1). The same questions were offered as written questionnaire in English and French for respondents unable to attend an interview.

Box 1 **Key informant interview questions.**

1 **Digital infrastructure** (e.g., access to internet, mobile phone availability, data affordability)

1.1 What opportunities have opened up in your country as a result of improved digital infrastructure?

1.2 How have improvements to digital infrastructure benefited the health system?

1.3 What are the outstanding gaps/problems when it comes to the digital infrastructure?

1.4 What steps has your country taken to improve the digital infrastructure in recent years?

2 **Digital health** (e.g., telemedicine, use of wearables, internet use for information seeking, artificial intelligence)

2.1 What are the most significant changes that you have observed in the area of digital health in recent years?

2.2 How have children and young people (below 25 years) benefited from these changes?

2.3 Where do you see the largest gaps and barriers in implementation of the national digital health strategy?

2.4 Are there further steps that you see your country taking to push for further digital transformation of the health system?

3 **Growing up in a digital world**

3.1 How are young people and children considered specifically in the digital (health) policies?

3.2 Are young people included in monitoring/implementation of digital health and policy development? If so, how?

3.3 How do you think digital health approaches could better support young people's health and wellbeing in the future?

4 **Governance of data in health**

4.1 Does your country have data protection laws in place with specific regard of health data?

4.2 Are there special governance and protection rules for children and young people?

4.3 What are the barriers to effective implementation?

In total, 18 individuals agreed to participate in an interview and 11 semi-structured interviews were successfully held in English using Zoom or Skype. A further two respondents provided written responses to the interview questions in French (see Table 8 for respondents' profiles). Interviews were conducted under Chatham House rules so that respondents felt able to speak freely and in their personal capacities. Interviews were recorded and transcribed. Interview and questionnaire data was extracted and compiled in a database. Data was manually coded using the rounded theory method.

**Table 8:** Profile of experts interviewed for the study.

**Expert location**	**Expert profile**	**Format of responses**
Cameroon	Civil society organization implementing digital health services	Interview
DRC	Former advisor to the government	Interview
East Africa (Regional expert)	International organization working with governments	Interview
Ethiopia	International organization working with the government	Interview
Mali	Civil society organization implementing digital health services	Questionnaire
Mali	Government official	Questionnaire
Nigeria	Civil society organization implementing digital health services	Interview
Nigeria	Private sector organization implementing digital health services	Interview
Tanzania	Government official	Interview
Tanzania	International organization working with the government	Interview
Uganda	Academic institution working with the government	Interview
Uganda	International organization working with the government	Interview
Uganda	International organization working with the government	Interview

Despite regular outreach and follow-up, it was not possible to secure interviews with the desired number and diversity group of experts from each of the 10 countries. Locating suitable interviewees and conducting interviews during the COVID-19 pandemic proved challenging. Whilst the findings from such a small sample size cannot claim to be representative of the 10 countries, we felt the data to be sufficient to identify common barriers to implementation of digital health strategies and to capture different perspectives on the utility of focusing on children and youth in future national digital health strategies.

### Barriers to Implementing National Digital Health Strategies

Major barriers to implementation described by interviewees fell into four broad categories: weak leadership and coordination; underinvestment in basic infrastructure; weak “infostructure” (18) (i.e., weak health information systems to support data sharing and interoperability); and insufficient engagement of stakeholders. Addressing all of these bottlenecks is necessary for countries to realize the goals of their national digital health strategies and to accelerate progress toward UHC and greater health equity.

Lack of involvement of diverse stakeholders in the development of digital health strategies was perceived by interviewees as contributing to their slow implementation in many countries (see Table 9). Outside of a small group of stakeholders already working closely with governments on digital health, interviewees indicated that few people are aware that such strategies exist, let alone what their aspirations are. Interviewees who were involved in the development of digital health strategies confirmed that children and youth had not been consulted as part of the strategy development process. The evidence gap about young people's behaviors and experiences in relation to digital health was acknowledged to be a reason for the lack of focus on children and youth within the strategies.

**Table 9:** Illustrative interview extracts on stakeholder engagement in the development of digital health strategies.

**Theme**	**Quote**
Awareness of national digital health strategies	“*I wasn't aware there was a digital health strategy until you contacted me. It looks good on paper but it hasn't been popularized and unlikely to have been developed through an inclusive process.”* (Cameroon)
Stakeholder consultation process	“*[There was] a lack of timely consultation of stakeholders in the digital health process in Mali.”* (Mali) “*As they started to create awareness of the national strategy, then there was some conversation of how actors can support the vision, and for the first time people were achieving and aiming for the same goal.”* (Tanzania)
Civil society engagement	“*Data is used for reporting up but isn't shared back with communities. Communities don't have access to the data and can't use it to understand the health challenges in their area and whether services are meeting their needs. Individuals need to have access to their own data and health information.”* (Ethiopia)

The failure to engage diverse population groups, including children and youth, in the development of digital health strategies has resulted in a lack of ownership among the wider health community and contributes to ongoing fragmentation. Private sector stakeholders, for example, despite being major players in digital health, make very little contributions to the development of these strategies and have very little incentives for adoption. Limited engagement of civil society in the strategy development process weakens the potential for independent monitoring and accountability efforts that could accelerate progress toward implementation.

### Focusing on Children and Youth in Future Health Strategies

Most interviewees noted that whilst young people are not specifically considered in digital health strategies, child and adolescent health are clearly articulated priorities within broader health strategies and policies. Several interviewees also noted the demographic distribution in Africa to be a strong indicator for the future growth and adoption of digital health technologies. Young people, namely those in urban areas, were seen to more easily adopt digital technology and prioritize the need for broadband access, mobile phones, and the electricity required to power them. Young people are also helping to educate older family members about digital health. Some of the interviewees shared examples of their own work with young people who are training in digital health and involved in the development of digital health solutions.

When asked whether future digital health strategies should have a stronger focus on children and youth, responses fell into two camps (see Table 10). One group felt that strategies should maintain a whole population approach and that looking at different population groups may lead to unnecessary complexity and even further fragmentation. Other interviewees felt that future digital health strategies should pay greater attention to the specific needs of children, adolescents, and youth, and involve them in the strategy development process. Interviewees noted that young people are both the main users of digital technologies and have particular health and developmental needs that can be positively and negatively impacted by digital transformations. Some argued that future strategies should recognize the risks that young people are exposed to through digital means and also their need for stronger protections in relation to their health data.

**Table 10:** Illustrative interview extracts on the inclusion of children and youth within future digital health strategies.

**Theme**	**Quote**
Supporting a stronger focus on young people in digital health strategies	“*[The] revised digital health strategy should have a focus on different population groups and justify why. Children, adolescents and youth would be important to include. There are ways that digital technologies can incentivize positive behaviors and they are more attracted to digital technologies. They can also be negatively influenced. We have seen the benefits of tech in the COVID response. Certain issues that young people can talk about using digital tech; they would exchange more. Benefits for self-management of HIV. More confidential sources of information.”* (Uganda)
Questioning an approach that would prioritize young people in digital health strategies	“*As far as digital health is concerned… it is a sector that needs to be non-dis- criminatory. … We are now trying to take a holistic view and ensure whatever is being done [in digital health] will impact a wide range of stakeholders including adolescents, maternal, young girls, everybody.” (Tanzania)*

Whilst interviewees were divided on the benefits of including a stronger focus on children and youth within the content of digital health strategies, there was general agreement that the involvement of children and youth would add value to future policymaking. More inclusive processes would result in digital health approaches and interventions that better meet the needs of children, youth, and other underserved groups, as well as to increasing individuals' knowledge of their digital rights and digital health opportunities. Interviewees expressed an interest, yet lack of capacity, to more meaningfully include civil society, communities, and young people in the development of future strategies.

## Developing Digital Health Strategies that Better Serve the Health Needs of Children and Youth in a Digital Age: Recommendations for Policymakers

Digital health governance incorporates both the governance of digital technologies and data for health and the governance of the ways in which broader digital transformations influence health outcomes. Our review of 10 digital health strategies suggests that policymakers in countries with large populations of young people are prioritizing the former whereas the role of digital transformations as determinants of health for children, youth, and other groups are underexplored.

Digital transformations offer new ways for countries to accelerate progress toward UHC and goals related to newborn, child, adolescent, and youth health. We recommend that digital health strategies should identify the ways in which digital transformations can positively or negatively impact the health and wellbeing of different populations, and the forms of cross-sectoral and multi-stakeholder collaboration required to amplify or mitigate them. Even in countries with lower levels of digital maturity, anticipatory governance is important to maximize the health benefits and decrease future risks associated with digital transformations. By taking a broad and more holistic approach to digital health governance, a stronger focus on children and youth would likely emerge in countries with both large and smaller populations of young people since this group is entitled to additional protection from digital harms and also stands to gain so much from the benefits of digital health.

To ensure effective digital health governance, it is essential to design and implement national digital health strategies that fully reflect the needs and voices of children and youth. We therefore recommend that future digital health strategies are developed through a more inclusive process that involves children, youth, and other marginalized groups. As enshrined in human rights instruments such as the UN Convention on the Rights of the Child and African Charter on the Rights and Welfare of the Child, young people have a right to be involved in decision-making that affects their lives. Given the health risks presented to young people by digital transformations, and the implications of digital health approaches on other aspects of their lives, it is important to involve children and youth in the development of future digital health strategies so that the strategies steer a course toward more equitable health futures. More inclusive approaches to policy development would not only produce digital health strategies that more accurately reflect the health needs and priorities of children, youth, and other vulnerable groups, they would also foster greater ownership and accountability.

Finally, we recommend that policymakers and their partners invest in mechanisms for ongoing engagement of children and youth in digital health governance that avoid tokenism. This will require building capacity and skills within relevant government departments to effectively work with children and youth from diverse backgrounds, as well as creating channels for young people's voices to inform and monitor policymaking. As recommended by the WHO in its guidance on *Youth-centered digital health interventions*, crafting meaningful health interventions for youth requires meaningfully engaging youth (8). This is particularly relevant for the governance of digital transformations because policymakers may not comprehend the unique and novel ways in which the social worlds of youth interact with digital technologies and their infrastructures. When reflecting on barriers to implementation, interviewees for this study referenced the need for greater youth engagement, but often within traditional models of governance such as maternal, newborn, child, and adolescent health programs. They indicated that a more open space, both in consultation and governance, needs to be ideologically and financially fostered to create long-term, meaningful child and youth engagement in digital health.

This review poses several questions that merit further exploration. Additional research is needed to test whether the hypotheses arising from this study—namely that broadening the scope of digital health strategies and more inclusive policy development processes—will produce strategies that more explicitly prioritize the needs and views of children and youth. Longer-term analysis of digital health strategy implementation should also be undertaken to better understand the barriers faced by countries in translating their digital health ambitions into reality, and the effectiveness of different approaches to digital health in realizing UHC and health equity for current and future generations of children and youth.

## Author Contributions

LH: conceptualization, formal analysis, investigation, and writing—original draft. RS: formal analysis and writing—original draft. NN and ES: writing—review and editing. CF: conceptualization, formal analysis, investigation, and writing—review and editing. All authors contributed to the article and approved the submitted version.

## Funding

The *Lancet* and *Financial Times* Commission on Governing Health Futures 2030 was funded by Children's Investment Fund Foundation, Foundation Botnar, GIZ, SDC, UNICEF (in-kind), and Wellcome Trust. There was no involvement by the funding sources in the conceptualization, writing, or submission of the manuscript.

## Conflict of Interest

NN is a Commissioner of the *Lancet* and *Financial Times* Commission on Governing Health Futures 2030. NN was employed by PharmAccess Foundation. LH, RS, CF, and ES are members of the Secretariat of the *Lancet* and *Financial Times* Commission on Governing Health Futures 2030. CF was employed by CPC Analytics.

## Publisher's Note

All claims expressed in this article are solely those of the authors and do not necessarily represent those of their affiliated organizations, or those of the publisher, the editors and the reviewers. Any product that may be evaluated in this article, or claim that may be made by its manufacturer, is not guaranteed or endorsed by the publisher.

## Footnotes

^1^Lancet and Financial Times Commission on Governing health futures 2030: Growing up in a digital world: https://www.governinghealthfutures2030.org/.

^2^WHO directory of eHealth policies: https://www.who.int/goe/policies/countries/en/.

^3^ICT Works compilation of African digital health strategies: https://www.ictworks.org/african-national-ehealth-strategy-policy.

^4^Principles for Digital Development: https://digitalprinciples.org/ (accessed September 1, 2021).
